# Supplementary material for: Karyotyping with amniotic fluid in 6,572 pregnant women and pregnancy outcomes——A single-center retrospective study
Source: PLoS One. 2025 May 20;20(5):e0324744. doi: 10.1371/journal.pone.0324744 (PMC12091734; doi:10.1371/journal.pone.0324744)
Supplement: S1 File — (ZIP) [file pone.0324744.s001.zip › supporting information/PLOS consent-form-chinese.pdf]

PLOS 期刊出版同意书

本人即签署人，同意公共科学图书馆（Public Library of Science，PLOS） 期刊刊登我或我未成年子女（请在下面注明的地方填上儿童的姓名）的照片、其它图像或类似内容、病历或家族病历。我已看见并阅读过将出版的资料。我已跟 ____________________， 即本文的作者，讨论过本同意书，并理解以下条款：

所有 PLOS 期刊均可以从互联网上免费获取 [1]。因此任何人都可以阅读已出版的内容。读者不限于医生，也包括记者和其他公众。

我明白并接受以下各条款：

虽然不会公开我的姓名，而且 PLOS 也会移除所有可以找出我真实身份的资料，但我理解要做到百分百匿名是不可能的，因为可能某些人仍然会认出我。

在评审的过程中，文章的文本内容可能会在风格、文法、连贯性和长度上有所变动。

根据 PLOS 使用的知识共享许可协议 [2] (the Creative Commons Attribution License) ，在 PLOS 期刊上出版的内容可以免费被转载及用于任何法律用途，包括翻译成不同语言和商业用途。我理解我不会从这些内容中获得任何金钱或特许费用，我对这些内容以后的商业用途不能作出任何索偿。

签署这张同意书不会剥夺我的隐私权。我可以在文章出版前的任何时间收回我的同意，可是一旦内容进入出版程序（文章已送交去印刷），我不能收回同意。

如果其他家属牵涉在内（例如在家族史中提及），我确认我已获得他们的同意（包括出版内容的认可）。

姓名 __________________________________

未成年儿童的姓名（假如同意书是关于他们的）：______________________________

日期 ________________

签名 _________________________________

作者 _________________________________

日期 ________________

签署 _________________________________

[1]PLOS 期刊： https://www.plos.org/publications

[2]CC-BY；假如需要最新或之前版本，请浏览 https://creativecommons.org/licenses/

请填妥这张表格，取得病人的签名，并将病历归档。

如果文章有提到病人的详细资料，请标明病人已同意将该内容出版。
