# Supplementary material for: Karyotyping with amniotic fluid in 6,572 pregnant women and pregnancy outcomes——A single-center retrospective study
Source: PLoS One. 2025 May 20;20(5):e0324744. doi: 10.1371/journal.pone.0324744 (PMC12091734; doi:10.1371/journal.pone.0324744)
Supplement: Attachment — Submitted filename: Response to Reviewers.docx [file pone.0324744.s004.docx]

February 18, 2025

Editorial Department of PLOS ONE

Dear Editor,

Thank you for the opportunity to revise our manuscript. We sincerely appreciate the editor and reviewers for their positive and constructive comments and suggestions on our manuscript entitled “Karyotyping with amniotic fluid in 6,572 pregnant women and pregnancy outcomes—A single-center retrospective study”(ID: PONE-D-24-30679). We thoroughly checked the references in accordance with the journal’s requirements to ensure completeness and accuracy.

We have carefully considered the comments and made revisions to the paper. Responses to the second reviewer‘s comments are provided separately, and the corresponding page numbers for the modifications in the revised manuscript can be found in the rightmost column of the response table below. Additionally, the revised paragraphs (or sentences) are highlighted in different colors.

The revised paragraphs based on REVIEWER#2 is labeled in yellow.

We would like to extend our sincere appreciation to you and the reviewers for your valuable comments on our paper. We look forward to your response.

Thank you and beat regards.

Sincerely,

Min Ren*

Department of Obstetrics, Affiliated Hospital of Southwest Medical University, Luzhou, Sichuan, China.

Response to Reviewer#2 (The revised paragraphs based on REVIEWER#2 is labeled in yellow. )

| No. | Reviewer comment | Author response | Change made |
| --- | --- | --- | --- |
| 1 | INTRODCUTION: Lines 78-79 is not clear. Lines 87-88 should be referenced. | Thank you for your valuable feedback. Regarding Lines 78-79, which were unclear, we have rephrased the original text to improve clarity.  Regarding lines 87-88, we have added the references as suggested. | Page 4, Line 78-80.  Page 4, Line 87-88; Page 25, Line 403-404. |
| 2 | METHODS: The authors should state their study design. Lines 128-129 is not clear. | Thank you for your valuable feedback. We have added a detailed description of the follow-up study design after Lines 128-129 to ensure that readers fully understand our research methods and processes. | Page 6-7, Line 128-139. |
| 3 | DICSUSSION: Line 212 should be ---amniocentesis had abnormal karyotypes--. | Thank you for your valuable feedback. We have made revisions to the original text. | Page 17, Line 232. |
| 4 | DICSUSSION: Lines 292-293 is not clear while 301-303 does not seem complete. The statement in Lines 306-308 is not clear, are the authors referring to newborns from their study, if so how did they follow up their developmental patterns because this is not mentioned in the results or methods. Line 318 - offspring should be offsprings, and in Line 320, confirm should be confirms. | Thank you for your valuable feedback. Regarding Lines 292-293, which were unclear, we have rephrased the original text to improve clarity. Regarding Lines 301-303, we revised the incomplete sentences to enhance accuracy. Regarding Lines 306-308, we would like to clarify that the newborns mentioned refer to those who participated in this study. To ensure the comprehensiveness and reliability of the data, we employed various follow-up methods, including face-to-face interviews, telephone follow-ups, and online questionnaires. During the follow-up, we paid special attention to families with fetuses having chromosomal abnormalities, asking detailed questions about the child’s development. This included the specifics of 'Well-Child Care' (Child Health Management), physical development, and any abnormalities in daily behavioral performance. We have supplemented the follow-up methods and content in the Methods section. This follow-up data enabled us to effectively assess the child’s developmental status. In the Results section, we have added findings based on the follow-up data to enhance the study’s completeness and rigor. However, as the study only followed up to 6 months postpartum, our observations of child development were limited to this period. This limitation has been explicitly noted in the Limitations section. Regarding Lines 318 and 320, we have made the changes to the original text as suggested. | Page 21, Line 312-314.  Page 21, Line 322-327.  Page 21, Line 329-331; Page 6-7, Line 128-145; Page 16, Line 220-226.  Page 22, Line 341.  Page 22, Line 343.  . |
