# Supplementary material for: Karyotyping with amniotic fluid in 6,572 pregnant women and pregnancy outcomes——A single-center retrospective study
Source: PLoS One. 2025 May 20;20(5):e0324744. doi: 10.1371/journal.pone.0324744 (PMC12091734; doi:10.1371/journal.pone.0324744)
Supplement: Attachment — Submitted filename: Response_to_Reviewers_auresp_3.docx [file pone.0324744.s005.docx]

April 2, 2025

Editorial Department of PLOS ONE

Dear Editor,

Thank you for the opportunity to revise our manuscript. We sincerely appreciate the editor and reviewers for their positive and constructive comments and suggestions on our manuscript entitled “Karyotyping with amniotic fluid in 6,572 pregnant women and pregnancy outcomes—A single-center retrospective study”(ID: PONE-D-24-30679). We thoroughly checked the references according to the journal‘s requirements to ensure completeness and accuracy.

We have carefully considered the comments and revised the paper. Responses to the fourth reviewer‘s comments are provided separately, and the corresponding page numbers for the modifications in the revised manuscript can be found in the rightmost column of the response table below. Additionally, the revised paragraphs (or sentences) are highlighted in different colors.

The revised paragraphs based on REVIEWER#4 is labeled in yellow.

We would like to extend our sincere appreciation to you and the reviewers for your valuable comments on our paper. We look forward to your response.

Thank you and best regards.

Sincerely,

Min Ren*

Department of Obstetrics, Affiliated Hospital of Southwest Medical University, Luzhou, Sichuan, China.

Response to Reviewer#4 (The revised paragraphs based on REVIEWER#4 is labeled in yellow. )

| No. | Reviewer comment | Author response | Change made |
| --- | --- | --- | --- |
| 1 | To reflect the technology development trend and enhance the depth of research, discussion can be added about CMA. CMA could enhance the detection of pathogenic copy number variations (CNVs), particularly for fetuses with ultrasound anomalies but normal karyotypes.. | Thank you for your valuable feedback. Following your recommendations, we have expanded the discussion of chromosomal microarray analysis (CMA) in the Discussion section. These additions enhance the comprehensiveness of our study and better highlight the evolving role of genomic technologies in prenatal diagnosis. Thank you again for your constructive guidance on this study. | Page 21, Line 318-333.  Page 24, Line 394.  Page 30, Line 515-523. |
| 2 | Line 71: "Birth defects are …… other adverse pregnancy outcomes." can be reduced to："Birth defects—defined as structural, functional, or metabolic abnormalities arising during embryogenesis—may lead to spontaneous abortion, stillbirth, or congenital malformations." | Thank you for your valuable feedback. We have revised the original text according to your suggestions to make the sentences more concise and precise. | Page 4, Line 71-73. |
| 3 | Line 32: In Abstract method after the last "karyotyping" missing end punctuation, should write as "karyotyping." | Thank you for your valuable feedback. We have made revisions to the original text. | Page 2, Line 32. |
| 4 | Line 301: "conduct a analysis", correctly expressed as "conduct an analysis". | Thank you for your valuable feedback. We have made revisions to the original text. | Page 20, Line 299. |
| 5 | Line 341: "offsprings" should write as "offspring" | Thank you for your valuable feedback. We have made revisions to the original text. | Page 22, Line 355. |
